# Cavity Preparation

**Published:** 1904-07

**Authors:** Henry C. Raymond

**Affiliations:** Detroit, Mich.


### CAVITY PREPARATION.

By Henry C. Raymond, D. D. S.,
Detroit, Mich.

(Written for *The American Journal of Dental Science.*)

It is not the intention of this short article to give a detailed description of cavity formation for porcelain fillings, as the writer has not had the time in which to prepare models for the illustratons which should accompany such a paper. But some remarks in general on the subject may not be out of place when it is considered what a vague idea many seem to have regarding it.

Some writers on porcelain inlay work have been far from clear in their remarks on the preparation of cavities. This along with some unfortunate remarks in society discussions, from which many have been led to believe that the cement used in the work is the all important factor in retaining inlays, has led many to think that the form of the cavity is of secondary consideration; the main thing being a good cement, by the use of which a piece of porcelain may be made to retain its position on almost any kind of a formation, ranging all the way from that of a saucer-shaped cavity to a flat surface. This has been a grave error, and has done much to bring about the many failures that have occurred in the use of porcelain, and has discouraged many who have attempted it.

The correct formation of the cavity for any of the so-called permanent filling materials is of the greatest importance, and this applies as much to the use of porcelain as to any other material.

The saucer-shaped cavity which has been so often spoken of has no place in porcelain work at all. A cavity for a porcelain inlay of the simplest kind should have a definite formation, a formation for porcelain work, and differing principally from the form of cavity used for gold and amalgam, in that it must be without undercuts or interlocking walls. With a saucer-shaped cavity or one without a decided form, it is almost impossible to set the inlay in its correct position, as the cement beneath causes it to roll around and slip out of place; but with the walls at the proper angles, and base or floor forming a decided seat, it can only go to one position. For this reason a retentive form should be given to the simplest cavities, by cutting the walls so that they form an abtuse angle with the floor of the cavity. It is not meant that a sharp angle should be made at the junction of wall and floor, as we would then have a corner into which it would be difficult to burnish the matrix, but that the direction of the walls with the floor should be that of an obtuse angle, without the definite point which would form a perfect angle. This obtuse angle formation of cavity walls applies largely to all cavities, with some slight modifications. Parallel walls are not necessary, and should never be made, as they might interfere with withdrawing the matrix; and when setting the inlay the cement is likely to pack at the bottom of the cavity, and prevent it from going home, or if the inlay does go to place it sinks slightly below the cavity margin, which is not the case when the walls are at an obtuse angle.

To give a decided seat to this class of cavities, i. e., those on the labial surfaces and those on the approximal surfaces of the incisors and cuspids not involving the incisal surface, a slight depression should be cut with a small round bur on the floor of the cavity, usually in the form of a triangle. This is especially necessary when such cavities are rather shallow, though all cavities should be cut deep enough to prevent reflection of cement through a too thin body of porcelain.

In forming compound cavities, the cutting to the point of extension for prevention should be largely carried out, though it may not be necessary to carry this so far as for gold, as caries is less likely to recur around a porcelain filling. Of course we should cut far enough so that there may be strong walls, well supported by dentine wherever possible, especially in bicuspids and molars.

In approximo incisal cavities have as decided a base or seat at the cervical portion as the tooth will permit, and in the superior teeth the lingual walls should be cut away in excess of the labial, to prevent possible displacement of the filling by the inferior teeth during mastication. In these cavities a step should be cut on the lingual surface near the incisal edge if the tooth is strong and thick enough at that point, if it is not the step may be cut in the middle third or nearer the cervix. In the bicuspids and molars also get as good a seat at the cervical wall as possible, and as broad a step extending from the approximal surface to the occlusal as size of cavity will permit. With cavities formed in this way with definite bases for the inlays to rest upon, and walls cut at the proper angles, there need be little fear of displacement, provided of course that we get a perfectly burnished matrix and use proper care in the setting. In short the aim is to get all the retention form possible in all cavities, especially where the force exerted during mastication is likely to cause dsplacement, keeping well in the mind the fact that the formation must be such that the matrix may be withdrawn without changing its form. There must be *no* undercuts of any kind and angles must not be too definite. Walls must diverge toward the margins at all points. This in large, compound cavities requires careful watching.

Too much care cannot be used in preparing cavity margins. There must be no outward bevel at all, as there is for gold, as there is then a thin, weak margin or feathered edge to the inlay, which if it does not fracture when it is being set is likely to do so very soon afterwards. The bevel should be inwards and whenever possible it is best to have the natural bevel which comes from a continuous straight line from the floor of the cavity to margin, instead of breaking the line by forming a more acute angle at the margin, which gives a thin edge to the porcelain, and is consequently its weak point. This may not cause material trouble in simple inlays, though with these, when setting, if too much force is used on one side before the inlay is down in place a fracture may occur, and a faulty margin results. But inlays on or extending into the occlusal surface of bicuspids and molars having weak margins are certain to fracture there when subjected to pressure from the opposing teeth during mastication. In these cases especially, cut straight from the enamel to the floor of the cavity, finishing with a perfect angle at the margin, without further bevel. There is then a strong body of porcelain extending right to the margin, and the weak point so frequently spoken of in this class of fillings is entirely eliminated.

The marginal lines should be cut in rounded and curved forms, without angles, in the anterior teeth at any rate. Though some writers advocate definite angles at the margins in their method of cavity formation, and others think that such angles aid in the retention of the inlay, and also add to its natural appearance, it is the writer's experience that decided angles are not necessary either within the cavity or at the margins. Such angles make it very difficult to perfectly burnish the matrix at those points and frequently result in perforations there; such perforation at any marginal point of course ruins the matrix. It is much easier to burnish over slightly rounded corners, and curved marginal lines show the joint less and form a more natural appearance than is possible with straight lines and angles.

All margins should be well defined, cut to a knife edge, yet as smooth as possible.

This matter of cavity preparation, though governed by certain mechanical principles, is also largely governed by good judgment, without the use of which we can hardly expect to attain success in anything we undertake.

The location of a tooth being operated upon, its bulk and strength, with the masticatory force exerted upon it, must all be taken into consideration in the formation of the cavity to receive the porcelain, the object being to obtain all the retention form the requirements of the case call for and the circumstances will permit.

### THE PAST, PRESENT AND FUTURE OF PORCELAIN INLAYS.

By Charles C. Patten, D. D. S., Boston.

(Written for *The American Journal of Dental Science.*)

